# Supplementary material for: Tissue-resident memory B cells augment local anti-cancer immunity via IgA
Source: bioRxiv. 2026 Aug 13:2026.08.08.743713. Preprint. [Version 1] doi: 10.64898/2026.08.08.743713 (PMC13640654; doi:10.64898/2026.08.08.743713)
Supplement: Supplementary file 1 [file media-1.pdf]

Supplementary Materials for

Tissue-resident memory B cells augment local anti-cancer immunity via IgA

Abrar Samiea[1,2,3], Roxanne Bahn-Bales[1,2,3], Julia Vanderstreet[1,2,3], Maryam Al-Ghezi[1,2,3], Lina Gao[3,4], Malia Rettig[1,2,3], Zihan Guo[1,3], Rashi Yadav[3], Daniel O. Herzig[3,5], Sandy H. Fang[3,5], Liana Tsikitis[3,5], Adel Kardosh[3,6], Lauren B. Rodda[7], Ferdinando Pucci[1,3], Wesley Y. Yu[8], Rebekka Duhen[3] and Joshua M. Moreau[1,2,3,9]*

[1]Department of Cell, Developmental & Cancer Biology, Oregon Health & Science University, Portland, Oregon, USA

[2]Division of Oncological Sciences, Oregon Health & Science University, Portland, Oregon, USA

[3]Knight Cancer Institute, Oregon Health & Science University, Portland, Oregon, USA

[4]Cancer Data Science Shared Resource, Oregon Health & Science University, Portland, Oregon, USA

[5]Division of Gastrointestinal and General Surgery, Oregon Health & Science University, Portland, Oregon, USA

[6]Division of Hematology/Medical Oncology, Oregon Health & Science University, Portland, Oregon, USA

[7]Department of Molecular Microbiology & Immunology, Oregon Health & Science University, Portland, Oregon, USA

[8]Division of Dermatology, Sharp-Rees-Stealy Medical Group, San Diego, CA, USA

[9]Department of Dermatology, Oregon Health & Science University, Portland, Oregon, USA

*Corresponding author. Email: moreauj@ohsu.edu

The PDF file includes:

Materials and Methods
Supplementary Text
Figs. S1 to S5
Table S1

Materials and Methods

Clinical samples

Patients with CRC or BCC undergoing surgical resection were recruited through the Department of Dermatology and collaborating surgical oncology services at Oregon Health and Science University (OHSU) and were enrolled under OHSU Review Board-approved protocols (00025603 and 00022198). All participants provided written informed consent for tissue and blood collection. Fresh tumor and patient-matched adjacent tissue were collected at the time of surgery. For BCC specimens, fresh 6-mm punch biopsies of tumor and adjacent skin were analyzed. Peripheral blood was collected, when available, from CRC patients in EDTA tubes. In addition, healthy donor blood was collected. Specimens and PBMCs were processed into single-cell suspensions as described below and either used immediately or cryopreserved.

Analysis of single-cell RNA sequencing

Published processed single-cell RNA-seq datasets with cell-type annotations from Ma *et al. (16)* were downloaded at Cancer B cell blueprint (http://pancancer.cn/B/). Downstream analysis was performed in R using Seurat 5.3.0 *(51)*.

B_{RM} signature generation. To generate a conserved B_{RM} transcriptional program from both human and mouse B_{RM} signatures (**Fig. 1C**), B_{RM} up-regulated genes identified in human upper airway single-cell RNA-seq data by Ramirez *et al. (14),* and the 200 top genes enriched in mouse lung B_{RM} from Tan *et al. (8)* after conversion to corresponding human genes were examined. Genes identified in at least two independent datasets were retained, resulting in an 80-gene B_{RM} signature comprising both genes conserved between human and mouse B_{RM} and genes reproducibly identified across multiple human B_{RM} datasets.

B_{RM} module score analysis across cancer types. To determine B_{RM} signature enrichment across cancer types (**Fig. 1E**), B_{RM} 80-gene signature module scores were analyzed using the published multicancer single-cell B cell atlas generated by Ma *et al. (16).* The integrated dataset comprised 474,718 B cells from 477 samples across 269 patients and donors spanning 20 cancer types, including head and neck squamous cell carcinoma (HNSC; n = 21), breast cancer (BRCA; n = 26), renal cell carcinoma (RCC; n = 6), lung cancer (LC; n = 29), colorectal adenocarcinoma (COAD; n = 78), hepatocellular carcinoma (LIHC; n = 15), thyroid carcinoma (THCA; n = 14), esophageal carcinoma (ESCA; n = 32), stomach adenocarcinoma (STAD; n = 7), ovarian cancer (OV; n = 4), pancreatic adenocarcinoma (PAAD; n = 3), cholangiocarcinoma (CHOL; n = 8), gallbladder carcinoma (GBC; n = 9), thymoma (THYM; n = 4), cervical squamous cell carcinoma and endocervical adenocarcinoma (CESC; n = 4), gastrointestinal stromal tumors (GIST; n = 2), neuroblastoma (NB; n = 3), bladder urothelial carcinoma (BLCA; n = 1), uterine corpus endometrial carcinoma (UCEC; n = 1), and cutaneous T cell lymphoma (CTCL; n = 2). Processed expression matrices and associated metadata, including tissue location, patient identifier, tumor type, treatment status, and sample origin, were obtained from the original publications where available. Cell clustering and annotations were retained as originally defined by Ma *et al. (16)*. Cancer types with fewer than two matched patient samples were excluded from downstream comparative analyses. The conserved B_{RM} 80-gene signature module scores in each cell were calculated using "Seurat::AddModuleScore()" function with default parameters (genes in the signature set were scored relatively to 100 random control genes in the same expression bin across 24 bins).

For each cancer type, mean module scores were calculated separately for tumor-associated and peripheral blood B cells from matched patients, which were used as the unit of analysis. Mean paired differences were calculated as tumor minus blood module score, such that positive values indicated increased B_{RM} signature enrichment in tumor-associated B cells relative to matched peripheral blood. Statistical significance within each cancer type was determined using paired two-tailed *t*-tests.

Pathway enrichment analysis. Canonical pathway enrichment analysis of the B_{RM} 80-gene signature was executed using QIAGEN Ingenuity Pathway Analysis (IPA). The B_{RM} gene list was against the Ingenuity Knowledge Base to identify significantly enriched canonical pathways. Pathways were ranked by adjusted *P* value, and the top enriched pathways were displayed by percentage pathway overlap, defined as the proportion of genes in each pathway represented in the B_{RM} signature.

***TCGA data analysis*.** The TCGA Toil re-computed expression data and patient metadata were obtained from the TCGA Pan-Cancer cohort through the UCSC Xena website (https://xenabrowser.net/). To model the predictive value of B_{RM} on the ten-year overall survival of cancer patients (Fig. 2I), B_{RM} 80-gene signature enrichment scores were calculated using the AUCell R package as previously described by Yang *et al. (15)*. Briefly, patients were stratified into B_{RM}-high and B_{RM}-low groups according to the median AUCell enrichment score within each cancer type (*52*), and then the total B cell abundance for each TCGA tumor sample was obtained. Cox proportional hazards models were then used to evaluate associations between B_{RM} signature enrichment and overall survival, and hazard ratios with 95% confidence intervals were reported for each cancer type independently, where HR less than 1 indicated association with improved survival and HR more than 1 represented association with worse survival. In addition, Pan-cancer associations were subsequently estimated by random-effects meta-analysis. Kaplan–Meier survival curves were generated using the survival and survminer R packages.

Immunotherapy datasets analysis. To explore of whether the enrichment of B_{RM} signature was associated with immunotherapy response (**Fig. 2J**), we collected two bulk RNA-seq datasets of melanoma patients receiving anti-PD1 therapy. We kept only samples from tumors prior to immunotherapy. For the Gide *et al.* cohort *(40,41)*, pre-treatment anti-PD1 samples were retained, and ipilimumab plus anti-PD1 samples were excluded, resulting in 41 samples, including 19 responders. For the Liu *et al.* cohort *(41)*, 121 pre-treatment melanoma samples were analyzed. B_{RM} 80-gene signature enrichment scores were calculated for each sample using AUCell. For survival analyses, samples were dichotomized into B_{RM}-high tumors, defined as those above the 75th percentile of B_{RM} signature enrichment, and all other tumors.

Mice

Mb1-CreER^{T2} (JAX 033026), Rosa26 Ai14 (JAX 007914), Rosa26 DTA (JAX 009669), C57BL6/J (JAX 000664), and IgA-/- (Cyagen C001394) mice were purchased from Jackson laboratory or Cyagen biosciences and maintained in our laboratory. HEL-specific BCR-transgenic Hy10 mice (CD45.1 C57BL/6), maintained hemizygous for heavy or light chain transgenes, and TCR-transgenic OT-II mice (CD90.1 C57BL/6) were kindly provided by Dr. Ferdinando Pucci (OHSU) and used for adoptive transfer experiments. 6-16 week-old wild type 45.2-C57BL/6 mice, *Mb1-CreER^{T2}; Rosa-DTA* mice, or IgA KO female mice were used for tumor growth experiments. All mice were maintained in a specific pathogen-free environment, and all procedures were approved by the OHSU Animal Care and Use Committee.

Tumor cell lines

Two murine tumor cell lines were used in this study: the oral epithelial squamous cell carcinoma cell line MOC2 engineered to express transmembrane model antigens (OVA, HEL, HEL-OVA, or DEL-OVA) *(31)*, and the melanoma cell line B16-MO4-OVA (Sigma, SCC420). MOC2 cells were cultured in E-medium at 37°C and 5% CO2 as previously described *(31)*. B16-MO4-OVA cells were cultured at 37 °C with 5% CO_2 in RPMI complete medium (RPMI-1640 supplemented with 10% FBS, 1× non-essential amino acids (GIBCO), 10 mM HEPES (GIBCO), 54 µM β-mercaptoethanol (Thermo Fisher Scientific), and 1 mg/ml Geneticin (G418; Sigma 345810)). For *in vivo* tumor challenge experiments, 5 X 10^5 - 7 X 10^5 MOC2 cells were injected intradermally (i.d.), whereas 5 X 10^5 B16-MO4-OVA cells were injected intravenously (i.v.) to establish the experimental lung metastasis model.

Extracellular vesicle (EVs) generation. For the generation of B_{RM} in the skin, purified transmembrane EVs were used for vaccination. EVs were purified from oral epithelial squamous cell carcinoma cell line MOC2 engineered to express transmembrane model antigens mOVA, mHEL, linked mHEL-mOVA or linked mDEL-mOVA as previously described *(31)*.

Vaccination

For intraperitoneal (i.p.) priming and induction of endogenous OVA- or HEL-specific immune cells, mice were immunized i.p. with 10 µg mHEL–mOVA EVs emulsified in complete Freund's adjuvant (CFA). For laser assisted epicutaneous vaccination, mice were anesthetized using inhaled 2% isoflurane in O2 during the microporation procedure, and dorsal flank hair was removed using depilatory cream at least 1 day before microporation. Skin microporation was performed using the P.L.E.A.S.E. laser device (Pantec Biosolutions) as previously described with modifications (*35*). Briefly, mice were positioned to expose the dorsal flank skin, and microporation was performed using the following settings: fluence, 11.9 J/cm²; pulse duration, 75 µs; repetition rate, 200 Hz; pulses per pore, 2; pore array size, 14 mm²; and pore density, 8%. Three microporated regions were generated per vaccination site. Following microporation, purified EVs expressing membrane-bound model antigens were diluted in sterile PBS without adjuvant and applied evenly onto the microporated skin surface. Mice received 15 µg EVs in 40 µL sterile PBS per flank distributed across the three microporated regions, with mHEL–mOVA, mDEL–mOVA, or mHEL EVs administered to one flank and mOVA EVs administered to the contralateral flank where indicated. The EV suspension was allowed to passively absorb into the microporated skin for approximately 10 min before mice were returned to their cages. A booster vaccination was administered 7 days after the initial vaccination, and tumor challenge was performed 14-35 days after vaccination as indicated in the experimental design. For intranasal vaccination, mice were anesthetized using inhaled 3% isoflurane with O2, and immunized as previously described with modifications *(11)*. Briefly, mice received intranasal administration of OVA antigen adjuvanted with Poly(I:C) in sterile PBS using a micropipette-based delivery method. Mice were positioned in a supine position at an approximate 60° incline, and the jaw was gently maintained in a closed position during inoculation to promote inhalation and prevent swallowing of the inoculum. Vaccines were delivered dropwise into alternating nares in a final volume of 40-60 µL. Mice were monitored until full recovery following immunization. In most experiments, mice received 50 µg OVA adjuvanted with 5–10 µg Poly(I:C), followed by a booster immunization 14 days later.

In vivo B cells and T cell depletion

To deplete CD4 and CD8 T cells, mice were treated intravenously with 300 µg each of anti-CD4 (GK1.5, BioXCell) and anti-CD8 (2.43, BioXCell) antibodies on days -4 and -1 before, and days +2 and +4 after tumor challenge. In addition, mice received 100 µg each of anti-CD4 and anti-CD8 antibodies intranasally on day -1 before challenge. T cell depletion efficiency was confirmed by flow cytometry where indicated. For inducible B cell depletion studies, Mb1-CreER^{T2};Rosa26-DTA mice received intraperitoneal tamoxifen (MilliporeSigma) dissolved in corn oil at 75 mg/kg body weight once every 24 hours for 5 consecutive days.

Adoptive transfer

For adoptive transfer experiments, B and T cells were isolated from spleens and lymph nodes of HY10 mice (CD45.1$^{+/+}$) and OT-II mice (CD90.1$^{+/+}$) mice, respectively. Cells were processed into single cell suspensions. Cell quantity and purity were confirmed by flow cytometry following enrichment using negative selection kits (STEMCELL Technologies). Purified Hy10 B cells (5×10^5) and OTII CD4 T cells (2.5×10^5) were washed with PBS, resuspended in sterile PBS, and intravenously transferred into recipient Bl6.CD45.2 mice. One day after adoptive transfer, recipient mice underwent unilateral epicutaneous vaccination with mDEL-mOVA or mDEL EVs as described in the epicutaneous vaccination section.

Tissue processing

Mouse tissues were processed into single-cell suspensions as previously described *(53)*. To distinguish non-circulating tissue populations from circulating cells, mice received intravenous injection of 2 µg fluorochrome-conjugated anti-CD45 antibody 3 min before euthanasia, where indicated. Lungs were perfused by cardiac injection with 10 mL cold PBS before tissue collection. Skin and tumors were harvested, minced into smaller fragments in PBS, and centrifuged at 500 g for 5 min. Tissue supernatants were collected before digestion and stored at -80˚C for downstream protein analysis. Human tumors and matched adjacent tissue samples were processed similarly into single-cell suspensions. Under sterile conditions, specimens were cut into small pieces and digested in RPMI-1640 medium in the presence of hyaluronidase at 0.5 mg/ml (Sigma, H6254), collagenase at 1 mg/ml (Sigma, C5138), DNase at 30 U/ml (Roche, 04536282001) and human serum albumin (MP Biomedicals, IC08823051) at 1.5% final concentration. Cells were digested for 1 hr at 37°C under agitation. Cell suspensions were filtered through a 70 µm filter. Single-cell suspensions were cryopreserved until further analysis. Whole blood was collected into EDTA-coated tubes. Red blood cells were lysed using ACK lysis buffer for 3 min on ice, washed with cold PBS, and filtered through 40-µm strainers before downstream flow cytometric analysis. In some cases, human PBMCs were purified from whole blood over a Ficoll-Paque PLUS (GE Healthcare) gradient, washed and cryopreserved prior to analysis.

Flow Cytometry

For antigen-specific B cell identification, biotinylated recombinant OVA or HEL proteins were tetramerized with fluorochrome-conjugated streptavidin. Single-cell suspensions from mouse tissues were incubated with tetramers and decoy reagents, followed by magnetic column enrichment before starting surface staining as previously described *(36)*. Single-cell suspensions from mice or human samples were subsequently incubated with Zombie UV or Zombie Yellow Fixable Viability dye (Biolegend) for 15 min on ice to exclude dead cells, followed by Fc receptor blockade using anti-CD16/32 antibody (2.4G2) for 5 min at 4°C. Cells were then

stained with fluorophore-conjugated antibodies (**Table S1**) for 25 min at 4°C in the presence of Fc receptor blockade. Following surface staining, cells were fixed and permeabilized using the Foxp3/Transcription Factor Staining Buffer Set (ThermoFisher Scientific) according to the manufacturer's instructions. Stained cells were acquired using an Aurora flow cytometer (Cytek Biosciences).

FluoroSpot

For FluoroSpot assays, multiscreen 96-well plates (MAHAS4510, Millipore) were coated overnight at 4˚C with 15 µg of purified His-tag in PBS. Wells were washed and coated overnight at 4˚C with 10 µg of purified His-EpCAM antigen (SinoBiological, 10694-H08H-UE) in PBS. Plates were then washed with PBS and blocked with complete RPMI medium. Single-cell suspensions of human tissues and PBMCs were prepared as described above. Cells were thawed and $CD19^+$ $CD69^+$ IgD^- and $CD69^-$ cells were sorted on a flow cytometer (BD Symphony S6 Sorter). Sorted cells were washed, diluted in complete RPMI medium, and cultured in CTL-Test B Medium containing resiquimod (R848), IL-2 (ImmunoSpot, CTL-mBPOLYS-200). Cells were incubated for 5 days at 5% 37˚C with 5% CO_2. 5 days post-incubation, cells and supernatants were collected. Supernatants were used for antibody reactivity array analysis (HuProt, v4.0, CDI LABS). Cells were then washed and plated onto EpCAM-coated FluoroSpot plates in complete RPMI medium and incubated overnight before plates were analyzed using the FluoroSpot human IgG or IgA Double-Color FluoroSpot kit according to manufacturer's instructions. Developed FluoroSpot plates were visualized and counted manually using the quickstart FluoroSpot guide package in Elispot CTL plate reader (Cellular Technology Ltd.).

ELISA

To detect HEL and OVA specific IgA or IgG, 96-well flat bottom microplates (Corning high binding) were coated overnight at 4˚C with recombinant HEL (10 µg/mL) or OVA (20 µg/mL) protein diluted in PBS. Plates were washed with PBS containing 0.05% Tween 20 and blocked for 1–2 h with PBS containing 5% FBS at room temperature. Tissue or tumor supernatant were followed by incubation for overnight at 4°C. After washing four times with PBS-Tween 20, bound antibodies were detected using horseradish peroxidase (HRP)-conjugated anti-mouse IgG or IgA (1:3000 dilution). After 1 hour incubation at room temperature, plates were washed and developed using 3,3',5,5'-tetramethylbenzidine (TMB) solution (eBioscience). Reactions were stopped with 1M hydrolic acid and absorbance were measured at 450 nm on a microplate reader.

Statistics and software

All flow cytometry plots and histograms were generated using FlowJo software (Version 10, Treestar). Statistical analyses were performed using GraphPad Prism (Version 11). The statistical significance of differences in mean values between groups were determined using unpaired or paired two-tailed Student's t test, or one-way or two-way ANOVA with appropriate correction or multiple-comparison testing, as specified in the figure legends. A *P*-value of less than 0.05 was considered significant.

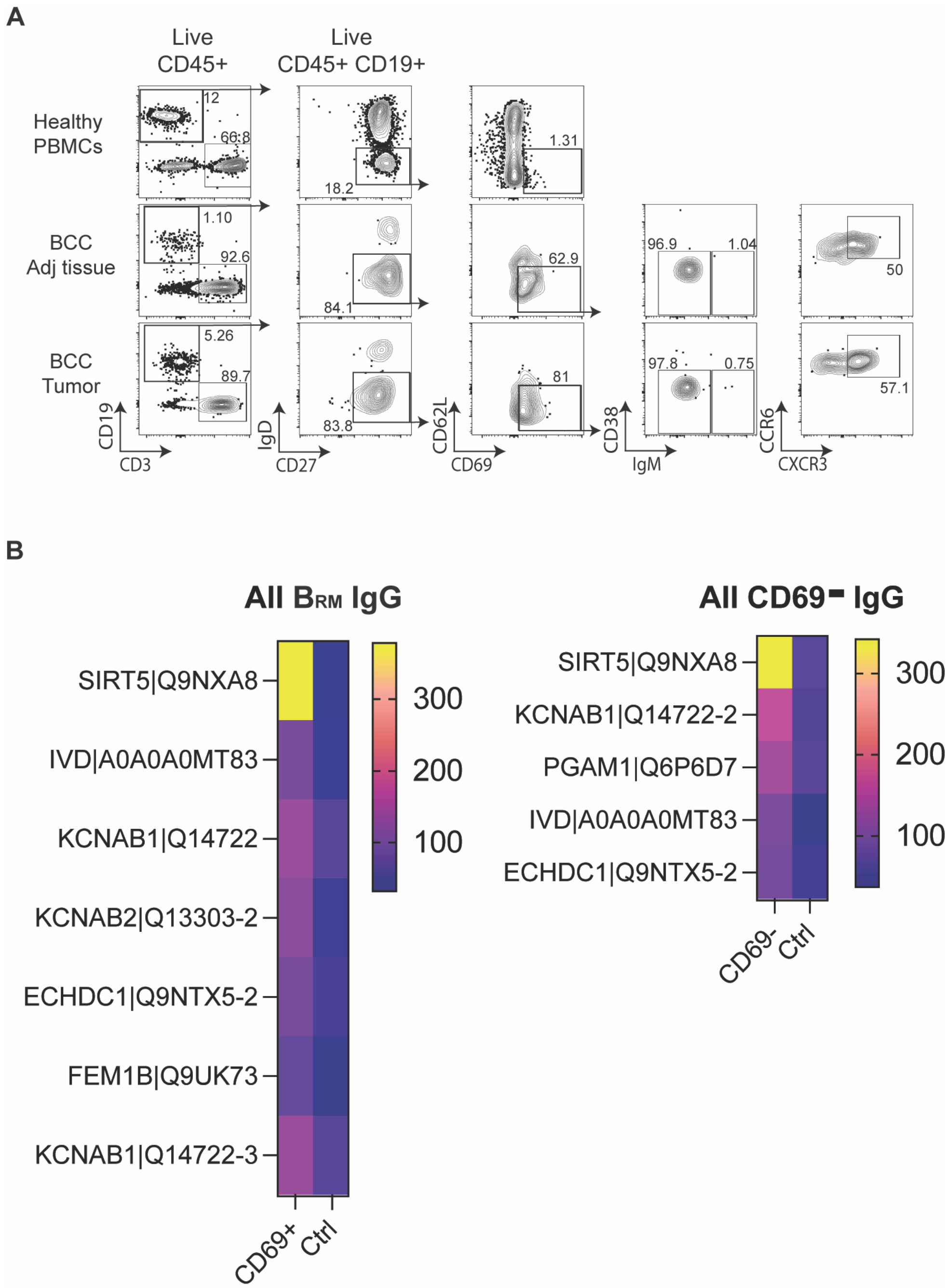

Fig. S1. Enrichment of B_{RM} cells in human tumors and adjacent tissues with autoreactivity. (**A**) Representative flow cytometry plots showing memory and class-switched B cells (IgD^- $CD27^+$) among live $CD45^+$ $CD19^+$ cells from BCC tumors, matched adjacent tissues, and healthy PBMCs. Memory B cells were further characterized for tissue-residency markers ($CD69^+$ $CD62L^–$), exclusion of germinal center and plasma cell phenotypes ($CD38^–$ $IgM^{-/+}$), and activation markers ($CCR6^+$ $CXCR3^+$). (**B**) Heatmaps showing the top antigen-specific IgG targets identified from culture supernatants of *in vitro*-differentiated live $CD45^+$ IgD^- $CD69^+$ B_{RM} (left) and $CD69^-$ B cells (right). Values represent normalized protein microarray signal intensities

relative to control samples, with color intensity indicating the magnitude of antigen-specific IgG reactivity.

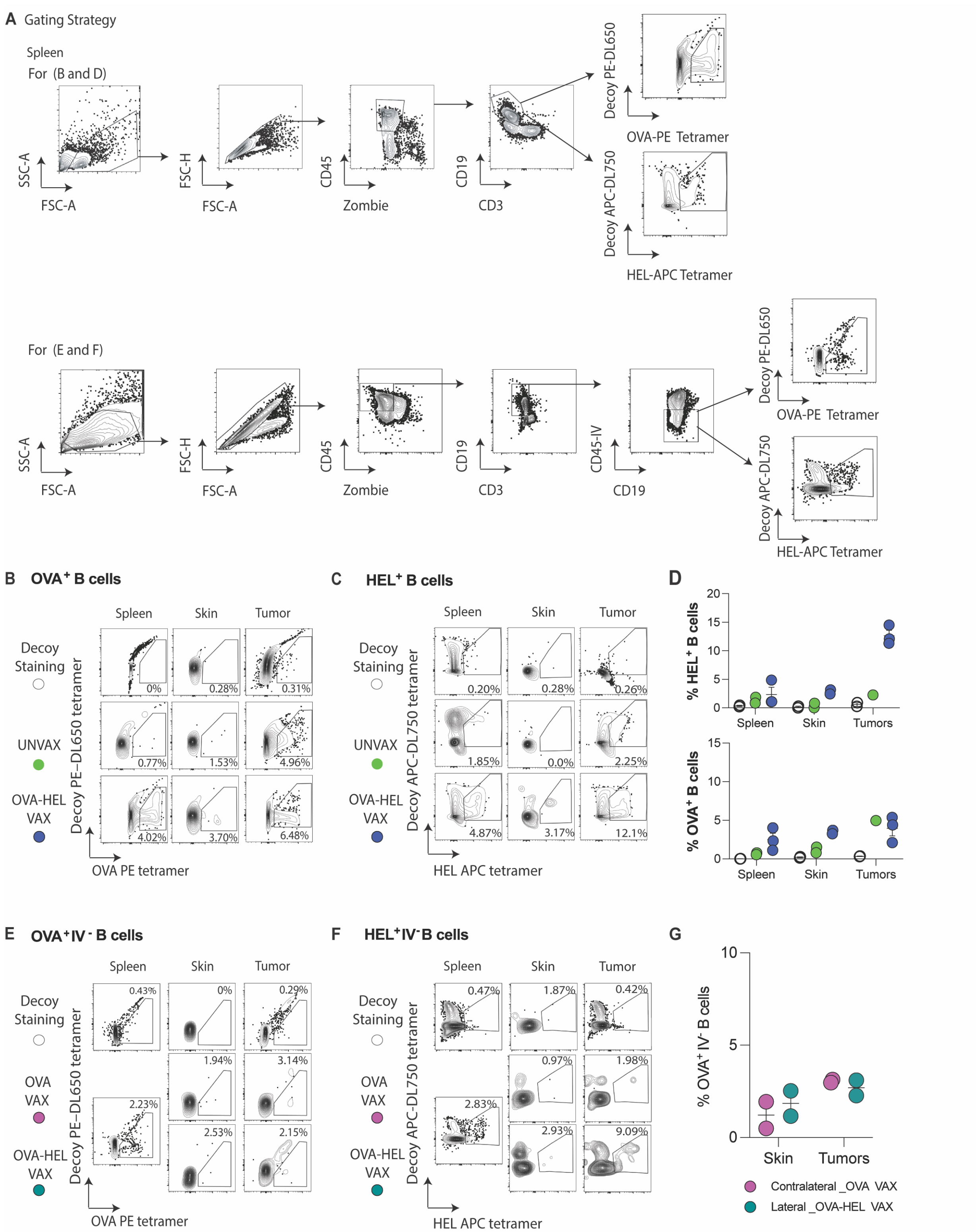

Fig. S2. HEL or OVA antigen-specific B cell identification in different tissues following local B$_{RM}$ generation. (**A**) Flow cytometry gating strategy with representative plots used to identify non-circulating HEL- or OVA- B cells from mouse spleen. Lineage exclusion included CD3ε staining followed by intravenous CD45 exclusion to identify non-circulating B cells.

Samples were acquired on a Cytek Aurora spectral flow cytometer. (**B** and **C**) Results obtained from gating in (**A**) to assess specific OVA or HEL B cells from spleen, skin and tumors of vaccinated C57BL/6 mice following the experimental strategy described in Fig. 1B. Skin tissues were collected 3 or 5 weeks after vaccination or mice were challenged intradermally with MOC2-mHEL tumor cells before collection of spleens and tumors to assess the presence of OVA (**B**) or HEL (**C**) specific B cells within vaccinated flanks. All harvested tissues were stained with decoy and tetramers. (**D**) Quantification of OVA-specific B cell frequencies is shown across indicated tissues. Results are pooled from two independent experiments. In each experiment, tissues from 5 mice were pooled, and each dot represents one pooled sample from an independent experiment.

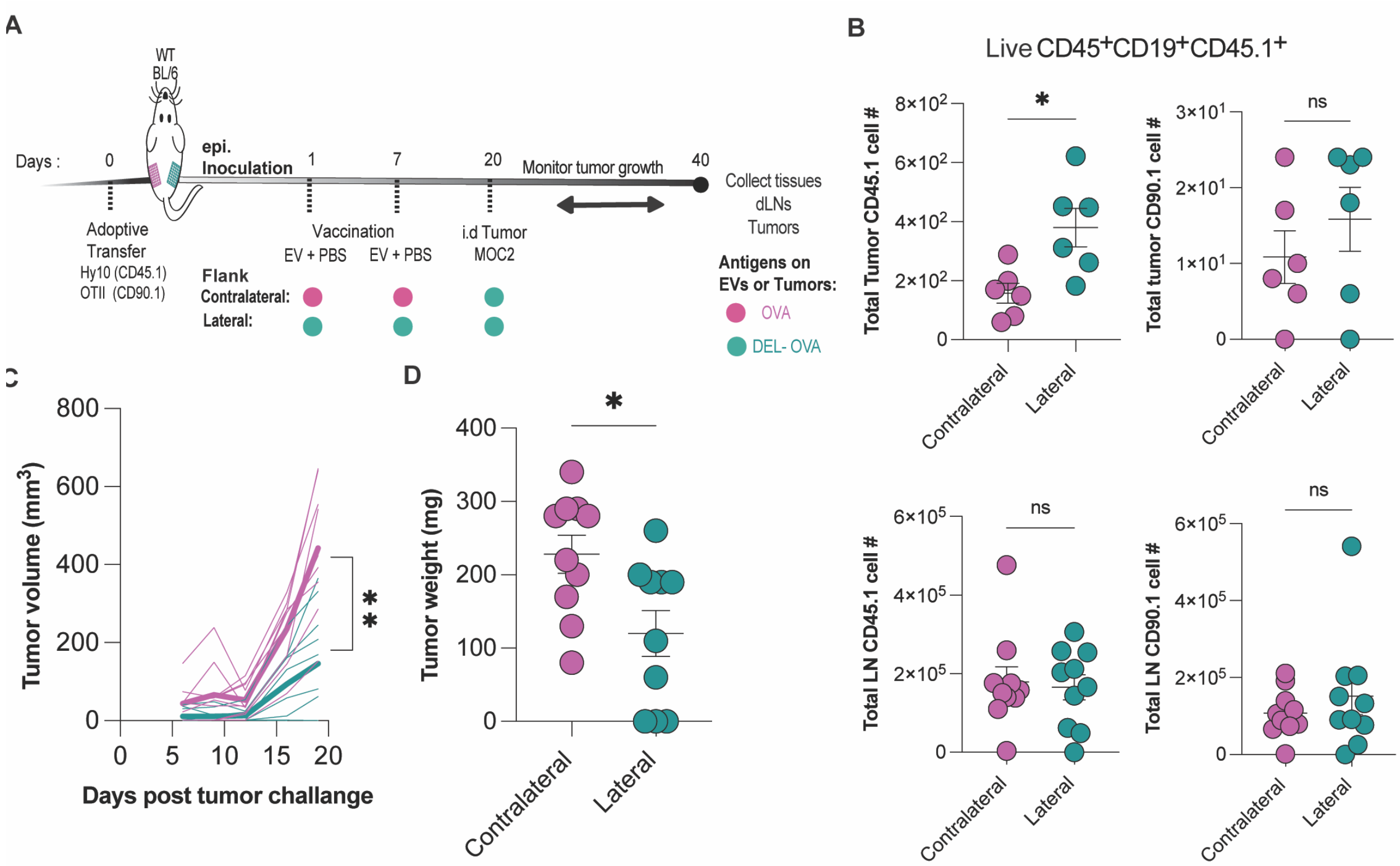

Fig. S3. Local B_{RM} established by Hy10 and OT-II cell adoptive transfer model augment anti-cancer immunity. (**A**) Schematic overview of the adoptive transfer, EV vaccination, and tumor challenge strategy. Hy10 B cells and OTII T cells were adoptively transferred into C57BL/6 recipient mice, followed by unilateral administration of EVs expressing model antigens and boosted with same agents 10 days later, as indicated. Two to three weeks post-vaccination, mice were challenged intradermally on both vaccinated flanks with 5×10^5 MOC2 tumor cells expressing linked mHEL–mOVA antigens. (**B**) Quantification of total Hy10 and OTII B cells in tumors and draining lymph nodes are shown following vaccination and tumor challenge. (**C**) Tumor growth kinetics following tumor implantation in both flanks (n = 10 mice). Data are presented as individuals or mean. (**D**) Tumor weights were measured at experimental endpoint. Paired tumor growth curves were analyzed using two-way ANOVA with Geisser-Greenhouse correction. Total Hy10 and OT-II B cells and endpoint tumor weights were analyzed using paired two-tailed *t*-test. $P < 0.05$ was considered statistically significant. Data are representative of two independent experiments.

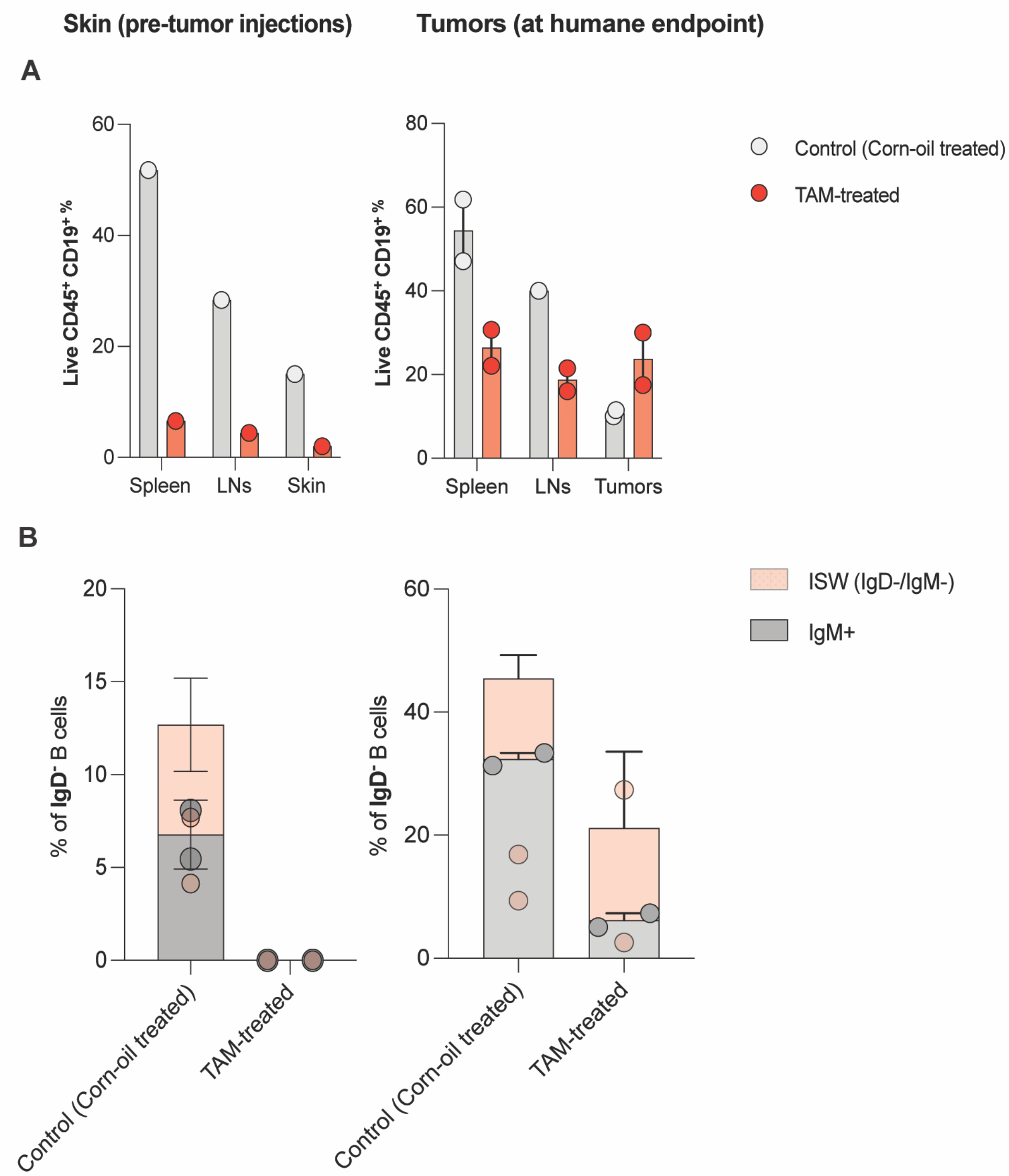

Fig. S4. Demonstration of effective inducible B cell depletion following local B_{RM} establishment. (**A**) Quantification of $CD19^+$ B cell frequencies among live $CD45^+$ B cells in spleen, draining lymph nodes (dLN), and skin from mb1-$CreER^{T2}$;Rosa26-DTA mice treated with tamoxifen (TAM) or corn oil control following local HEL-OVA specific B_{RM} establishment pre- and post- tumor challenge. (**B**) Quantification of class-switched (IgD^-IgM^-) and IgM^+ memory B cell populations in the skin and tumors of TAM-treated and control-treated mice (Skin; control n = 2 mice, in each indicated treatment group; 2 mice were pooled together); (Tumors; n= 2 mice in each indicated treatment group without pooling). Data are representative of one independent experiment.

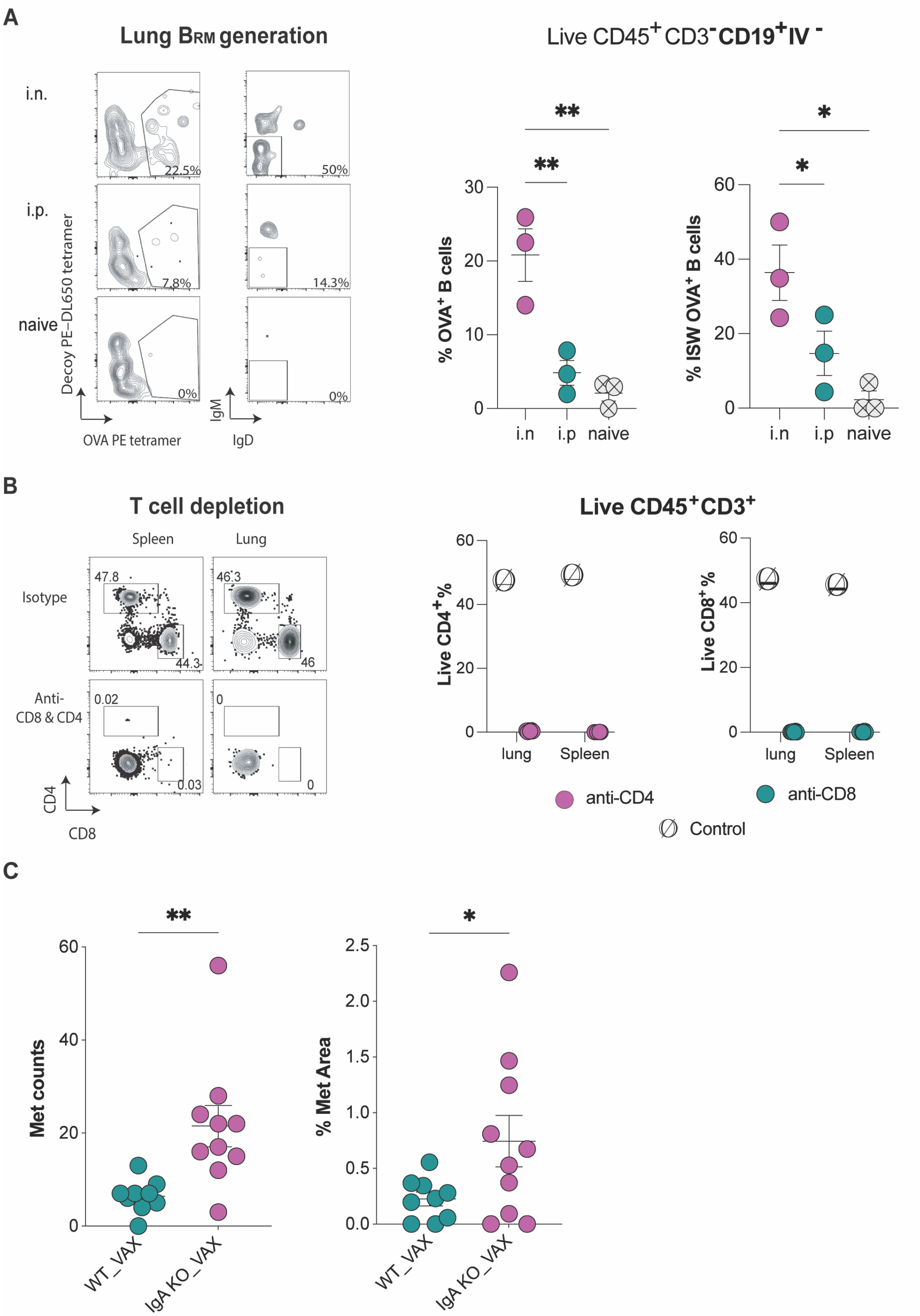

Fig. S5. Demonstration of effective lung BRM establishment and T cell depletion. (A and B) C57BL/6 mice were vaccinated intranasally (i.n.) or intraperitoneally (i.p.) with 50 µg OVA

adjuvanted with 5 µg Poly(I:C) and boosted 14 days later with the same agents. **(A)** Representative flow plots and quantification of OVA-specific B cells and class-switched (IgD^- IgM^-) OVA-specific B cells among non-circulating B cells from mouse lungs following vaccination. Gating strategy was adapted from Fig. S1A. Results are pooled from three independent experiments. In each experiment, lungs from two mice were pooled, and each dot represents one pooled sample from an independent experiment. (**B**) CD4 T and CD8 T cells depletion following intravenous and intranasal administration of anti-CD4 (GK1.5) and anti-CD8 (2.43) antibodies 25 days after vaccination. T cell depletion and quantification of CD4 T and CD8 T cells were assessed by flow cytometry. Data are representative of one independent experiment (isotype, n = 1; T cell depletion, n = 6). (**C**) IgA-deficient and wild-type control mice were either left unvaccinated or vaccinated intranasally (i.n.) with OVA antigen combined with and 5 µg Poly (I:C) and boosted with same agents before tumor challenge without T cell depletion (WT: VAX, n=9; $IgA^{-/-}$: VAX, n=10). Pulmonary metastatic burden was quantified by the number of metastatic foci and metastatic area. Data are presented as mean ± s.e.m. and are representative of one independent experiment. Comparisons between vaccinated IgA-deficient and wild-type mice were analyzed using unpaired one-tailed Welch's *t*-test.

Table S1. Antibodies

**Antibodies**	Source	Product ID
Flow cytometry: anti-mouse CD3 17A2 Spark Blue 574	Biolegend	100249
Flow cytometry: anti-mouse CD45 30-F11 APC/Cyanine7	Biolegend	103115
Flow cytometry: anti-mouse CD273 (PDL2) TY25 BUV563	BD Biosciences	741431
Flow cytometry: anti-mouse CD138 281-2 PE/Fire640	Biolegend	142543
Flow cytometry: anti-mouse IgM RMM-1 BV711	Biolegend	406539
Flow cytometry: anti-mouse CD45R RA3-6B2 BV510	BioLegend	103248
Flow cytometry: anti-mouse CD19 6D5 APC/Fire 810	Biolegend	115577
Flow cytometry: anti-mouse CD80 MP5-20F3 Alexa Fluor® 488	BD Biosciences	561363
Flow cytometry: anti-mouse CD73 TY/11.8 NovaFluor™ Yellow 730	Thermo Fisher	M032T02Y07
Flow cytometry: anti-mouse CD69 H1.2F3 Spark NIR 685	Biolegend	104557
Flow cytometry: anti-mouse CXCR3 (CD183) CXCR3-173 NovaFluor™ Blue 610-70S	Thermo Fisher	M037T03B06
Flow cytometry: anti-mouse CD279 (PD1) 29F.1A12 PE fire 810	Biolegend	135253
Flow cytometry: anti-mouse IgD AMS 9.1 BV480	BD Biosciences	746452
Flow cytometry: anti-mouse CD38 90 PerCP/Cyanine5.5	Biolegend	102722
Flow cytometry: anti-mouse IgA IC10-1 BUV395	BD Biosciences	743299
Flow cytometry: anti-mouse CD44 IM7 BUV661	BD Biosciences	741471
Flow cytometry: anti-mouse CD43 S7 BUV737	BD Biosciences	612840
Flow cytometry: anti-mouse CD62L MEL-14 BUV805	BD Biosciences	741924
Flow cytometry: anti-mouse CD103 20000000 Alexa Fluor® 594	Biolegend	121428
Flow cytometry: anti-mouse Ly6G 1A8 Spark Blue 550	Biolegend	127663
Flow cytometry: anti-mouse TCR γ/δ GL3 BV605	Biolegend	118129
Flow cytometry: anti-mouse CD3 17A2 BV750	Biolegend	100249
Flow cytometry: anti-mouse CD8a RPA-T8 BV570	Biolegend	301037
Flow cytometry: anti-mouse CD4 GK1.5 APC	Biolegend	100411
Flow cytometry: anti-mouse CD25 PC61 BV650	Biolegend	102038
Flow cytometry: anti-mouse CD11c HL3 BUV737	BD Biosciences	612797
Flow cytometry: anti-mouse CD11b M1/70 BV750	BioLegend	101267
Flow cytometry: anti-mouse CD11a M17/4 BUV496	BD Biosciences	741071
Flow cytometry: anti-mouse CD196 (CCR7) 29-2L17 BV785	Biolegend	129823
Flow cytometry: anti-mouse CD5 53-7.3 PE-Cy5	BioLegend	100610
Flow cytometry: anti-mouse CD45.1 A20 BV421	BD Biosciences	563983
Flow cytometry: anti-mouse TIM1 RMT1-4 PE	BioLegend	119506
Flow cytometry: anti-mouse CD274 (B7-H1, PD-L1) 10F.9G2 PE/Dazzle™ 594	BioLegend	124324
Flow cytometry: anti-mouse TCRb H57-597 Alexa Fluor® 700	BioLegend	109224
Flow cytometry: anti-mouse CD19 6D5 APC-Fire 810	BioLegend	115578
Flow cytometry: anti-mouse CD73 TY/11.8 PE-Dazzle 594	BioLegend	127234

Flow cytometry: anti-mouse CD69 H1.2F3 Spark NIR 685	BioLegend	104558
Flow cytometry: anti-mouse CD183 (CXCR3) CXCR3-173 BV650	BioLegend	126531
Flow cytometry: anti-mouse Ly-6G 1A8 Spark Blue 550	Biolegend	127664
Flow cytometry: anti-mouse CD8a 53-6.7 BV570	BioLegend	100740
Flow cytometry: anti-mouse CD4 RM4-4 BV605	BioLegend	116027
Flow cytometry: anti-mouse CD80 16-10A1 BV421	BD Biosciences	562611
Flow cytometry: anti-mouse CD138 281-2(RUO) BB515	BD Biosciences	566207
Flow cytometry: anti-mouse CD43 S7 BV750	BioLegend	747277
Flow cytometry: anti-mouse CD45 30-F11 PerCP-Cy5.5	BioLegend	103131
Flow cytometry: anti-mouse GL7 GL-7 (GL7) eFluor 450	Thermo Fischer	48-5902-82
Flow cytometry: anti-mouse CD38 90 PE-Fire 700	BioLegend	102747
Flow cytometry: anti-mouse IgG GOT214A BUV615	BD Biosciences	752758
Flow cytometry: anti-mouse TCR b H57-597 BV510	BioLegend	109234
Flow cytometry: anti-mouse CD4 Ultra Violet GK1.5	BD Biosciences	613006
Flow cytometry: anti-mouse CD80 Violet 16-10A1	BioLegend	104725
Flow cytometry: anti-mouse IgG Violet Poly4053	BioLegend	405327
Flow cytometry: anti-mouse CD3 Blue 17A2	BioLegend	100276
Flow cytometry: anti-mouse ki67 Yellow-Green 11F6	Biolegend	151218
Flow cytometry: anti-mouse FoxP3 Blue MF-14	Biolegend	126405
Flow cytometry: anti-mouse CD45R RA3-6B2 AF700	Biolegend	103231
Flow cytometry: anti-mouse IgD 11-26c.2a BV510	Biolegend	405723
Flow cytometry: anti-mouse F40/80 BM8 BV785	Biolegend	123141
Flow cytometry: anti-mouse CD45.2 104 (RUO) PerCP/Cyanine5.5	BD Biosciences	552950
Flow cytometry: anti-mouse CD45R (B220) AF 700 RA3-6B2	BioLegend	103232
IV Labeling for Flow cytometry: anti-mouse CD45 I3/2.3 FITC	Biolegend	147710
IV Labeling for Flow cytometry: anti-mouse CD45 30-F11 (RUO) APC R700	BD Biosciences	565478
Invivo T Cell Depletion: anti-mouse CD4 GK1.5	BioXCell	#BE0003-1
Invivo T Cell Depletion: anti-mouse CD8 2.43	BioXCell	#BE0061
Flow cytometry: anti-humanCD45 BUV395 HI30	BD Biosciences	563791
Flow cytometry: anti-humanViability Zombie UV [N/A]	Biolegend	423108
Flow cytometry: anti-humanCD45RA BUV563 HI100	BD Biosciences	612926
Flow cytometry: anti-humanIgM BUV661 UCH-B1	BD Biosciences	750365
Flow cytometry: anti-humanCD69 BUV737 FN50	BD Biosciences	564439
Flow cytometry: anti-humanCD80 BUV805 L307.4	BD Biosciences	742046
Flow cytometry: anti-humanCD279 (PD-1) BV421 NAT105	Biolegend	367422
Flow cytometry: anti-humanCD196 (CCR6) Pacific Blue G034E3	Biolegend	353439
Flow cytometry: anti-humanCD11c BV480 B-ly6	BD Biosciences	566135
Flow cytometry: anti-humanCD62L BV510 DREG-56	Biolegend	304844
Flow cytometry: anti-humanCD8 BV570 RPA-T8	Biolegend	301038

Flow cytometry: anti-humanIgD BV605 IA6-2	Biolegend	348232
Flow cytometry: anti-humanCD183 (CXCR3) BV650 G025H7	Biolegend	353730
Flow cytometry: anti-humanCD24 BV711 ML5	Biolegend	311136
Flow cytometry: anti-humanCD185 (CXCR5) BV750 J252D4	Biolegend	356942
Flow cytometry: anti-humanCD138 BV786 MI15	BD Biosciences	743501
Flow cytometry: anti-humanT-bet Alexa Fluor 488 525831	R&D	FAB53851G
Flow cytometry: anti-humanCD3 Spark Blue 550 SK7	Biolegend	344852
Flow cytometry: anti-humanCD27 NovaFluor Blue 610-70S O323	ThermoFisher	H012T03B06-A
Flow cytometry: anti-humanCD4 PerCp RPA-T4	Biolegend	300528
Flow cytometry: anti-humanCD273 (PDL2) PerCP-Cy5.5 MIH18	BD Biosciences	564256
Flow cytometry: anti-humanCD307d (FcRL4) PE 413D12	Biolegend	340204
Flow cytometry: anti-humanCD197 (CCR7) PE-Dazzle 594 G043H7	Biolegend	353236
Flow cytometry: anti-humanCD19 PE-Cy5 HIB19	Biolegend	302209
Flow cytometry: anti-humanCD103 PE/Fire 700 Ber-ACT8	Biolegend	350240
Flow cytometry: anti-humanCD25 PE-Cy7 BC96	Biolegend	302612
Flow cytometry: anti-humanCD307e (FcRL5) APC 509f6	Biolegend	340306
Flow cytometry: anti-human Foxp3 Alexa Fluor 700 PCH101	ThermoFisher	56-4776-41
Flow cytometry: anti-humanCD38 APC/Fire 750 HB-7	Biolegend	356626
Flow cytometry: anti-humanCD73 NovaFluor Red 755 AD2	ThermoFisher	H016T03R06-A
FluoroSpot: anti-human CD45 BUV395 HI30	BD Biosciences	563791
FluoroSpot: anti-human CD8 BUV805 SK1	BD Biosciences	564913
FluoroSpot: anti-human CD3 BV510 OKT3	Biolegend	317331
FluoroSpot: anti-human IgD BV605 IA6-2	Biolegend	348231
FluoroSpot: anti-human CD4 AF488 RPA-T4	Biolegend	300519
FluoroSpot: anti-human CD19 PE-Cy5 HIB19	Biolegend	302209
FluoroSpot: anti-human CD27 APC M-T271	Biolegend	356409
FluoroSpot: anti-human CD45 BV711 HI30	BioLegend	304050
FluoroSpot: anti-human CD19 PE HIB19	Biolegend	302208
FluoroSpot: anti-human CD69 APC-R700 FN50	BD Biosciences	565154
Cell sorting: anti-human CD45 HI30 BV711	BioLegend	304050
Cell sorting: anti-human CD19 HIB19 APC-Fire 810	BioLegend	302271
Cell sorting: anti-human CD69 FN50 Spark NIR 685	BioLegend	310957
Cell sorting: anti-human IgD IA6-2 BV480	BD Horizon	566138
Cell sorting: anti-human CD3 OKT3 eFluor 450	Invitrogen	48-0037-42
